# Association of Preoperative Serum Magnesium Levels and Acute Kidney Injury Following Thromboembolectomy in Acute Limb Ischemia

**DOI:** 10.5152/eurasianjmed.2026.251340

**Published:** 2026-05-27

**Authors:** Mesut Engin, Ahmet Kağan As, Ufuk Aydın, Yusuf Ata, Şenol Yavuz

**Affiliations:** Department of Cardiovascular Surgery, Bursa Yuksek Ihtisas Training and Research Hospital, University of Health Sciences, Bursa, Turkey

**Keywords:** Lower extremity ischemia, magnesium, renal injury, revascularization, risk factor

## Abstract

**Background:**

A critical cardiovascular emergency is acute lower extremity ischemia (ALI). Acute kidney injury (AKI) after surgical embolectomy prolongs hospitalization and increases treatment costs. Low blood magnesium (Mg) levels were shown to be associated with renal dysfunction. In this study, the goal was to investigate the connection between AKI and preoperative Mg levels.

**Methods:**

Patients who underwent a surgical embolectomy for ALI between January 2016 and June 2024 were consecutively included in this retrospective study. After the exclusion criteria, patients were divided into 2 groups: those who developed AKI in the postoperative period (group 1) and those who did not (group 2).

**Results:**

The median ages of the 403 patients in group 2 and the 69 patients in group 1 were 64 (39-95 years) and 67 (35-89 years), respectively (*P *= .098). There was no statistical difference in terms of gender, prior cerebrovascular events, hypertension, diabetes mellitus, hyperlipidemia, rates of coronary artery disease, and peripheral arterial disease between the 2 groups. Glycated hemoglobin >9 (OR: 1.350, CI 95%: 1.110-1.984, *P *= .028), high creatinine (OR: 2.945, CI 95%: 1.837-3.682, *P *< .001), and low Mg (OR: 0.695, CI 95%: 0.550-0.869, *P *= .014) values were determined as independent predictors for AKI.

**Conclusion:**

The current study demonstrates that preoperative low Mg levels may be a risk factor for AKI.

**Main Points**

- A critical cardiovascular emergency is acute lower extremity ischemia that threatens lower extremity survival. Acute kidney injury (AKI) after surgical embolectomy prolongs hospitalization and increases treatment costs.
- In the literature, various blood parameters affecting the development of AKI after acute cardiovascular interventions have been investigated.
- Magnesium (Mg) is a very important cation for human cells. It contributes to regulating neural functions in the body, stabilization of nucleic acid structures, and enzyme activation and acts as a catalyst in many biochemical events
- The current study demonstrates that preoperative low magnesium levels are an independent predictor of AKI risk.
- These situation needs to be supported by prospective multicenter studies. If supported by these studies as well, Mg replacement may be performed in patient groups considered at risk.

## Introduction

A critical cardiovascular emergency is acute lower extremity ischemia (ALI) that threatens lower extremity survival. The most significant outcomes in these patients are certainly mortality and limb loss; however, complications such as infection and acute kidney injury (AKI) may also occur.^1^

Ischemia-reperfusion injury occurs after acute surgical embolectomy due to muscle breakdown. Then, a rapid inflammatory reaction is brought on by the release of chemokines (Monocyte chemotactic protein [MCP], interleukin-8, etc.) and inflammatory-promoting cytokines. Thus, the risk of renal damage increases. In addition, AKI after surgical embolectomy prolongs hospitalization and increases treatment costs. In addition to resulting in permanent renal damage, this condition is an important cause of mortality; therefore, patients should be closely monitored for renal function in the postoperative period.^2^

In the literature, various blood parameters affecting the development of AKI after acute cardiovascular interventions have been investigated.^3^^,^^4^ One of these values is magnesium (Mg). It is a crucial cation for human cells. It helps stabilize nucleic acid structures, activates enzymes, controls the body’s neurological processes, and serves as a catalyst for numerous metabolic reactions.^5^^,^^6^ In 1 study in this field, preoperative low blood Mg levels were found to be associated with AKI after cardiac surgery.^5^ In another study, it was concluded that low blood Mg levels were associated with renal dysfunction after primary coronary percutaneous intervention.^6^

In line with this recent literature information, the goal was to investigate the connection between preoperative Mg levels and AKI, which is a significant complication that occurs after surgical embolectomy.

## Material and Methods

Patients who underwent a surgical embolectomy for ALI between January 2016 and June 2024 were consecutively included in this retrospective study. Demographic characteristics, preoperative blood values, and operative and postoperative data were acquired from the hospital information system and patient files. Patients with hypercoagulation diseases, oncologic diseases, diabetic foot ulcers, Buerger’s disease, hemodialysis patients, Rutherford class III patients, and patients with active infection were all excluded. Acute kidney injury was accepted as an elevation in serum creatinine of at least 1.5 times or a 0.3 mg/dL rise in serum creatinine within 72 hours after the surgical embolectomy.^7^ After the exclusion criteria, 98 patients were excluded. Then, 472 consecutive patients were included in the study, and patients were split into 2 groups: those who developed AKI in the postoperative period (group 1) and those who did not (group 2). Before their operations, the patients provided written informed consent. The University of Health Sciences, Bursa Yuksek Ihtisas Training and Research Hospital ethics committee approved the study, which was conducted in compliance with the Declaration of Helsinki (2024-TBEK 2025/07-06).

Blood parameter evaluations of patients were performed using samples taken from peripheral veins during hospital admission. The diagnosis and treatment strategy for ALI in the clinic have been previously described.^8^

### Acute Lower Extremity Ischemia Diagnosis and Treatment Plan

All patients suspected of ALI have a thorough history recorded and a physical examination conducted. The suspicion of ALI is also supported by Doppler ultrasonography. These tests enable the identification of ALI and the planning of surgical treatment. Computed tomographic angiography imaging was employed in certain patients. Following diagnosis, every patient was immediately admitted to the intensive care unit and underwent surgery. All patients received light sedation and a local anesthetic. After a linear incision in the femoral region, the common femoral, superficial femoral, and profunda femoral arteries were twisted and suspended. A Fogarty catheter (3-7 French) was used to perform an embolectomy following the arteriotomy to the common femoral artery. When sufficient distal and proximal flow was obtained, the arteriotomy was primarily healed. The process was repeated until the thrombus material ceased to protrude. Every patient was closely monitored in the intensive care unit for at least a day following the procedure. Hourly heart rate checks were conducted during this time. In medical therapies, a low molecular weight heparin (1 mg/kg, sc) treatment was administered for at least 1 week following the heparin infusion on the first day (the activated clotting time remained constant at 200-250 s). After the first day, clopidogrel 75 mg/day and 100 mg/day of acetylsalicylic acid were administered in addition to these therapies.

### Statistical Analysis

Data analysis was performed using IBM SPSS Statistics for Windows (IBM Corp., 2012, Version 21.0, 70 Armonk, NY: IBM Corp.). For numerical data, the normality of the distribution was evaluated using the Shapiro–Wilk and Kolmogorov–Smirnov tests. For data that was not normally distributed, the Mann–Whitney *U*-test was utilized; for data that was normally distributed, the Student’s *t*-test was employed. The mean, standard deviation (SD), and median (minimum-maximum) values were computed for numerical data. The percentage and frequency values were computed for categorical data, and the Chi-square test was utilized to analyze them. Firstly, the univariate logistic regression analysis was applied to reveal the predictors of postoperative AKI. Variables whose *P* value in univariate analyses was less than .05 were included in the multivariate logistic regression analysis. To forecast AKI, the area under the curve (AUC) was computed using receiver-operating characteristic (ROC) curve analysis for serum Mg levels. *P* < .05 was used to determine the test results’ statistical significance.

## Results

The study covered about 472 consecutive ALI patients. The median ages of the 403 patients in group 2 and the 69 patients in group 1 were 64 (39-95) and 67 (35-89), respectively (*P *= .098). The history of prior cerebrovascular events, gender, hypertension, diabetes mellitus (DM), hyperlipidemia, rates of peripheral and coronary artery disease, atrial fibrillation, and current smoking rates were all similar across the 2 groups. The percentage of patients in group 1 with Rutherford class IIB and first symptom development to admission times longer than 6 hours was higher (*P *= .029, *P* = .019, respectively). Vascular occlusion areas of the 2 groups were similar (Table 1).

Two groups were similar in terms of white blood cell, hematocrit, platelet, HbA1c, sodium, potassium, and calcium levels. Preoperative basal creatinine value was considerably greater in group 1 than in group 2 (*P* < .001). Preoperative serum Mg and albumin levels were significantly higher in group 2 (*P *< .001, *P* = .009, respectively) (Table 2).

The factors influencing AKI following surgical embolectomy operations were identified using binary logistic regression analysis. In univariate analysis, age > 70 (OR: 1.195, 95% CI: 1.190-2.180, *P* = .019), Rutherford class IIb (OR: 1.435, 95% CI: 1.195-1.894, *P* = .018), HbA1c > 9 (OR: 1.562, 95% CI: 1.272-2.190, *P* = .007), low albumin (OR: 0.594, 95% CI: 0.485-0.817, *P* = .013), high creatinine (OR: 4.242, 95% CI: 3.156-6.549, *P* < .001), and low Mg (OR: 0.796, 95% CI: 0.585-0.898, *P* < .001) values were found to be significantly correlated with the development of AKI. As a result of the multivariate analysis, HbA1c > 9 (OR: 1.350, CI 95%: 1.110-1.984, *P *= .028), high creatinine (OR: 2.945, CI 95%: 1.837-3.682, *P* < .001) and low Mg (OR: 0.695, CI 95%: 0.550-0.869, *P* = .014) values were determined as independent predictors for predicting AKI (Table 3).

Another multivariate logistic regression analysis was utilized to predict the occurrence of postoperative AKI according to risky preoperative creatinine and Mg levels. As a result of the analysis, “Creatinine >2mg/dL and Mg <1.79 mg/dL” situation (OR: 8.945, CI 95%: 6.451-11.385, *P *< .001) was determined as a strong independent predictor for predicting AKI (Table 4).

**Figure 1:**
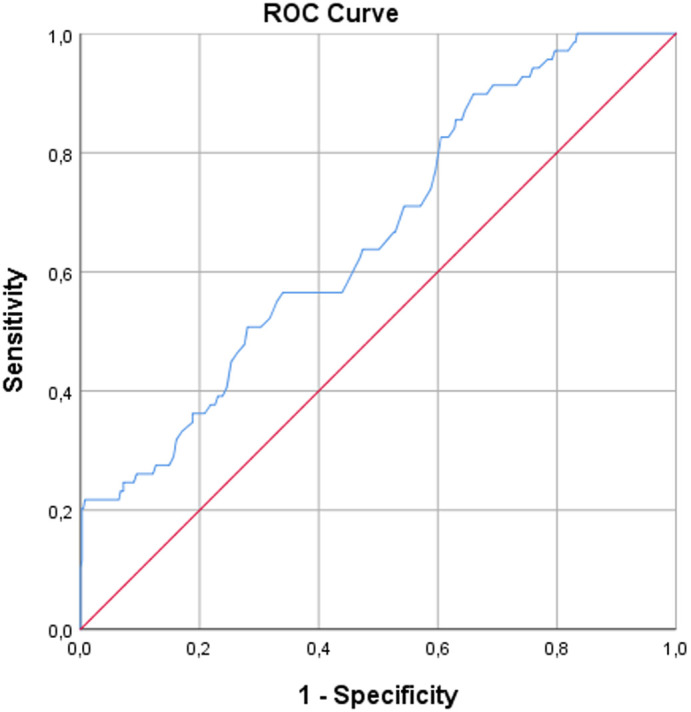
Receiver-operating characteristic curve and area under the curve for serum magnesium level for predicting postoperative acute kidney injury (cut-off: 1.79, AUC: 0.660, 95% CI: 0.592-0.728, *P* < .001, 63.8% sensitivity and 51.4% specificity).

The ROC curve analysis was used to evaluate how well Mg levels predicted AKI after surgical thromboembolectomy procedures. The cut-off value of Mg was 1.79 (AUC: 0.660, 95% CI: 0.592-0.728, *P *< .001, with 63.8% sensitivity and 51.4% specificity) (Figure 1).

## Discussion

The current study concluded that in addition to known AKI risk factors, preoperative low serum Mg levels may increase the risk of postoperative AKI in patients undergoing surgical embolectomy.

With advancing age, renal reserve decreases due to structural and physiological changes in the kidneys. Therefore, elderly patients are more vulnerable to renal complications in their health concerns.^9^ Kubat et al^2^ investigated clinical outcomes after surgical embolectomy in ALI patients. In this study, the AKI rate was approximately 11%. When the patients were analyzed as those above and below 80 years of age, 7% more AKI was detected in elderly patients. In the study, the AKI rate was found to be 14.6%. In the multivariate analysis, being over 70 years of age was significantly correlated with the risk of AKI (OR = 1.295, *P* = .019). In a recent study involving a large cohort of patients, factors affecting the AKI development following surgery were investigated. At the end of the study, in addition to comorbidities such as hypertension and DM, advanced age was shown as a predictor of AKI.^10^ Another recent study indicated that patients with ALI had worse clinical outcomes when they were older.^11^

Patients who develop ALI may be affected by the ischemic process at different levels. The most commonly used classification in terms of the possibility of reversibility and risk of amputation in these patients is the Rutherford ALI classification, denoted as Rutherford I, IIA, IIb, and III, whereas Class III is considered irreversible and Class IIb indicates a very critical condition.^12^ Thus, the conditions in this clinical classification indicate the severity of the ischemic event in the lower extremity tissue. When blood circulation is provided to severely ischemic tissue, ischemia-reperfusion injury occurs. The resulting free radicals can affect all organs, especially the kidneys.^13^ In the current study, it was found that a significant positive correlation between patients with Rutherford stage IIB and the development of AKI (OR = 1.435, *P* = .021). Another study has also shown a significant correlation between the severity of ischemia and AKI.^14^

Diabetes mellitus is a significant risk factor in the development of cardiovascular diseases and plays an important role in prognosis. Diabetes mellitus is also an important cause of AKI in the general population due to renal microvascular problems.^15^ In the study, the presence of uncontrolled DM (HbA1c >9%) was an independent predictor of AKI (OR = 1.350, *P *= .028). In a study conducted in the neurovascular field, high HbA1c values were found to be significant in predicting the risk of AKI after endovascular treatment in patients with ischemic stroke.^16^ In another study conducted by Kocogullari, a significant association between high HbA1c values and postoperative AKI was found in patients without DM, undergoing coronary bypass surgery.^17^ It was also shown that there was a correlation between the incidence of AKI and high HbA1c values in patients undergoing revascularization after acute myocardial infarction.^18^

Human serum albumin is a significant blood protein that functions as a main drug binder and exerts oncotic pressure, which controls fluid transfers across bodily compartments and impacts blood flow to the kidneys.^19^ A meta-analysis by Hansrivijit et al^20^ found that low albumin levels were associated with the development of AKI in hospitalized patients. Arora et al^21^ investigated the AKI risk factors in a study of 684 patients with chronic leg ischemia who underwent revascularization. The rate of AKI in the postoperative period was found to be 12%. At the conclusion of their study, the authors found that an albumin level below 3 g/dL was an independent predictor of AKI (OR = 1.66, *P* < .001). Unlike this study, however, patients with ALI were included, and surgical embolectomy was performed in all patients. In this study, it was found that an AKI rate of 14.6% and a significant negative association between albumin levels and AKI (OR = 0.594, *P* = .013).

AKI, which is seen between 2 and 18% of all hospitalized patients, can occur at rates close to 60% in intensive care unit patients. One of the most important reasons for the occurrence of this condition is high preoperative creatinine.^22^ In a study, preoperative creatinine elevation was shown to be an important predictor of AKI risk after coronary bypass surgery.^23^ In the study, preoperative creatinine elevation was the strongest predictor of postoperative AKI risk (OR = 2.945, *P *< .001). In another cardiovascular study, baseline renal function was shown to be the strongest predictor of postoperative AKI.^24^ In the present study, a strong predictor of AKI was identified in the multivariate analysis by combining a preoperative high creatinine value and low Mg value (Table 4).

Magnesium is a very important cation for human cells. It contributes to regulating neural functions in the body, stabilization of nucleic acid structures, enzyme activation and acts as a catalyst in many biochemical events. Therefore, many complications may arise from Mg deficiency. In a study involving 16 082 patients with malignancy, risk factors for AKI after hospitalization were investigated, and hypomagnesemia was found to be an important predictor of AKI.^25^ In the cardiovascular field, Koh et al^5^ investigated the risk factors for AKI after cardiac surgery. At the end of the study, preoperative serum Mg levels below 1.09 mg/dL were found to be an important predictor of AKI. Again, in early 2025, the impact of preoperative Mg levels on contrast nephropathy in patients receiving coronary intervention was examined by Demirtola et al^6^ As a result of multivariate analysis, Mg2+ levels below 2.03 mg/dL were shown to be an independent predictor for the development of contrast nephropathy. The association between serum Mg levels and postoperative mortality and morbidity in patients undergoing emergency peripheral vascular surgery was examined by Whittaker et al^26^ In a study of 197 patients, low serum Mg levels were shown to be an independent predictor of early mortality and cardiac complications.

The study’s primary weakness is that it was a retrospective single-center study, which made it unable to assess AKI indicators like cystatin C, a significant predictor of kidney impairment. The AKI assessment was based on the determined increase in creatinine levels at admission. Urine-output criteria were not used. Furthermore, due to the retrospective design of the study, some variables [Operative time, blood loss/transfusion, nephrotoxic medications (NSAIDs, aminoglycosides, ACEi/ARB), or markers of rhabdomyolysis (CK, myoglobin)] that can affect kidney function could not be included in the analyses. Apart from this, amputation and postoperative major cardiovascular analysis evaluations could not be performed in the study. In addition, the presence of coronary artery disease was evaluated according to the history of previous coronary intervention and coronary bypass. Because the coronary angiography results for the entire patient population were unavailable, some patients may have been overlooked.

Acute kidney injury after vascular surgical operations is an important problem due to the associated prolonged hospitalization and mortal consequences. Therefore, it is imperative to identify the risk factors and to take precautions whenever possible. The current study demonstrates that preoperative low Mg levels are an independent predictor of AKI risk. These situations need to be supported by prospective multicenter studies. If supported by these studies as well, Mg replacement may be performed in patient groups considered at risk

## Figures and Tables

**Table 1.** Demographic Data of the Patients

**Variables**	**Group 1 ** **(N = 69)**	**Group 2 ** **(N = 403)**	***P***
Age (years)	67 (35-89)	64 (39-95)	.098^ ‡^
Male gender, n (%)	43 (62.3)	255 (62.3)	.879^*^
Diabetes mellitus, n (%)	19 (27.5)	78 (19.4%)	.120^*^
Hypertension, n (%)	36 (52.2)	249 (61.8)	.131^*^
Hyperlipidemia, n (%)	23 (33.3)	119 (29.5)	.524^*^
CAD, n (%)	27 (39.1)	141 (35)	.507^*^
PAD, n (%)	17 (24.6)	128 (31.8)	.236^*^
Smoking, n (%)	15 (21.7)	99 (24.6)	.612^*^
Previous CVE, n (%)	5 (7.2)	18 (4.5)	.322^*^
Atrial fibrillation, n (%)	6 (8.7)	25 (6.2)	.440^*^
CT Angiography, n (%)	45 (65.2)	229 (56.8)	.192
FSD to A time >6 hours (%)	14 (20.3)	42 (10.4)	.019^*^
Occlusion region			.589^*^
Iliofemoral occlusion, n (%)	47 (68.1)	261 (64.8)	–
Femoropoliteal occlusion, n (%)	22 (31.9)	142 (35.2)	–
Rutherford class, (%) I IIA IIB	10 (14.5) 26 (37.7) 33 (47.8)	91 (22.6) 184 (45.7) 128 (31.8)	.029^*^

CAD, coronary artery disease; COPD, chronic obstructive pulmonary disease; CT, computed tomography; CVE, cerebrovascular event; FSDtoA, first symptom development to admission; PAD, peripheral arterial disease.

^*^Chi-square test.

^‡^Mann–Whitney *U*-test.

**Table 2.** Preoperative Laboratory Test Results and the Patients’ Perioperative Characteristics

**Variables**	**Group 1** **(N = 69)**	**Group 2** **(N = 403)**	***P***
White blood Cell (10^3^/µL)	9.9 (5.9-17.6)	9.5 (6.6-18.1)	.291^‡^
Hematocrit (%)	37 (27-55.1)	38 (28-53.6)	.646^‡^
Platelet (10^3^/µL)	248 (100-450)	246 (138-445)	.519^‡^
Creatinine, mg/dL	1.5 (0.8-3.8)	1.2 (0.7-2.9)	<.001^‡^
Albumin (g/L)	35 (29-50)	41 (32-48)	.009^‡^
Na (mEq/L)	137.9 ± 2.16	138.1 ± 2.21	.375^t^
K (mEq/L)	4.15 ± 0.53	4.14 ± 0.55	.742^t^
Ca (mg/dL)	9.09 ± 0.48	9.12 ± 0.5	.641^t^
Mg (mg/dL)	1.6 (1.2-2.6)	2.1 (1.6-2.9)	<.001^‡^
HbA1c, %	5.5 (4.8-15)	5.7 (4.5-11)	.119^‡^

HbA1c, hemoglobin A1c; K, potassium; Mg, magnesium; Na, sodium.

^t^Student T test .

‡Mann–Whitney *U*-test.

**Table 3.** Univariate and Multivariate Logistic Regression Analysis for Acute Kidney Injury Risk

	**Univariate Analysis **			**Multivariate Analysis **		
**Variables**	***P***	**Exp(B) Odds** **Ratio**	**95% CI** **Lower-Upper**	***P***	**Exp(B) Odds ** **Ratio**	**95% CI ** **Lower Upper**
Age >70 years	.019	1.295	1.190-2.180	.138	1.135	0.790-1.785
Rutherford class IIb	.021	1.435	1.195-1.894	.265	1.380	0.885-2.140
Diabetes mellitus	.134	1.114	0.860-1.255	–	–	–
HbA1c >9%	.007	1.562	1.272-2.190	.028	1.350	1.110-1.984
Albumin, mg/dL	.013	0.594	0.485-0.817	.334	0.892	0.664-1.190
Creatinine, mg/dL	<.001	4.242	3.156-6.549	<.001	2.945	1.837-3.682
Mg mg/dL	<.001	0.796	0.585-0.898	.014	0.695	0.550-0.869

HbA1c, glycated hemoglobin A1c; Mg, magnesium.

**Table 4.** Multivariate Logistic Regression Analysis According to Risky Preoperative Creatinine and Magnesium Levels for Acute Kidney Injury Risk

	**Multivariate Analysis**		
**Variables**	***P***	**Exp(B) Odds ** **Ratio**	**95% CI ** **Lower-Upper**
Age >70 years	.295	1.058	0.790-1.230
Rutherford class IIb	.346	1.035	0.895-1.686
Albumin, mg/dL	.442	0.796	0.690-1.090
HbA1c >9%	.104	1.060	0.890-1.350
Creatinine >2mg/dL and Mg <1.79 mg/dL	<.001	8.945	6.451-11.385

## Data Availability

The data that support the findings of this study are available on request from the corresponding author.
